# Ensemble Learning-Based Feature Selection for Phage Protein Prediction

**DOI:** 10.3389/fmicb.2022.932661

**Published:** 2022-07-15

**Authors:** Songbo Liu, Chengmin Cui, Huipeng Chen, Tong Liu

**Affiliations:** ^1^School of Computer Science and Technology, Harbin Institute of Technology, Harbin, China; ^2^Beijing Institute of Control Engineering, China Academy of Space Technology, Beijing, China

**Keywords:** machine learning, ensemble learning, feature selection, phage, protein classification

## Abstract

Phage has high specificity for its host recognition. As a natural enemy of bacteria, it has been used to treat super bacteria many times. Identifying phage proteins from the original sequence is very important for understanding the relationship between phage and host bacteria and developing new antimicrobial agents. However, traditional experimental methods are both expensive and time-consuming. In this study, an ensemble learning-based feature selection method is proposed to find important features for phage protein identification. The method uses four types of protein sequence-derived features, quantifies the importance of each feature by adding perturbations to the features to influence the results, and finally splices the important features among the four types of features. In addition, we analyzed the selected features and their biological significance.

## Introduction

Phages, which are the most abundant and widespread organisms on the Earth, can replicate within and destroy the host cell. Phages play an important role in microbial physiology, population dynamics, evolution, and therapy (Clokie et al., 2011), affecting biochemical systems worldwide (Jahn et al., 2019).

Phages also influence the development of anti-cancer drugs. The use of phages to target cancer cells for a specific binding for therapeutic purposes has been applied in clinical trials for cancer treatment due to differences in surface markers of tumor cells from normal cells (Yu et al., 2021). The identification of phage proteins is important for understanding the relationship between phages and host bacteria and for developing novel drugs or antibiotics (Lekunberri et al., 2017), and therefore, thorough investigations must be performed to identify the specific components recognized by phages.

Traditional physical experimental methods such as mass spectrometry (MS), sodium dodecyl sulfate polyacrylamide gel electrophoresis (SDS-PAGE), and protein arrays (Lavigne et al., 2009; Yuan and Gao, 2016; Jara-Acevedo et al., 2018), which have been used to identify phage viral proteins, are expensive and often time-consuming. Traditional biological methods such as cell separation, electron microscopy, and fluorescence microscopy are less feasible for analyzing large-scale biological data (Mei, 2012; Li et al., 2015). Computational models can not only analyze large amounts of biological data but also make preliminary predictions of unknown protein sequences, which is an excellent complement to traditional experimental methods.

In recent years, protein function prediction has been a hot topic in the field of computational biology (Ding et al., 2020; Fu et al., 2020; Guo et al., 2020). With the increasing amount of protein data, the techniques of applying machine learning and data mining to protein function prediction have gradually matured (Liu et al., 2019; Zhao et al., 2021). Several researchers have used machine learning to predict the function of protein sequences through sequence analysis (Chou, 2009; Cui et al., 2019; Jin et al., 2021), position-specific scoring matrix (PSSM) (Jones, 1999), various physicochemical and biochemical properties of amino acids, sequence conservation, amino acid composition, domain interactions, and geometrical and biophysical properties (Kawashima and Kanehisa, 2000; Cai et al., 2003; Zulfiqar et al., 2021). Feng et al. (2013) developed a Naive Bayes-based model for protein classification, which used amino acid composition (AAC) and dipeptide combination (DPC) as input features. Ding et al. (2014) developed a support vector machine (SVM) prediction model. In this method, analysis of variance was applied to select significant features from the g-gap DPC. Recently, Zhang et al. (2015) developed a random forest classification method to distinguish phage virus protein (PVP) from non-PVP. A novel feature extraction method with a two-layered structure is proposed (Xiong et al., 2018; Jiang et al., 2021). First, the features irrelevant to the results are removed by the filter or wrapper method, and then, the results of the previous step are used in the model for classification and prediction.

Since each feature extraction method extracts only part of the protein sequence information, the method of combining multiple protein information for classification is proposed in the absence of a clear sequence or structural information. Jiao and Du (2017) proposed the functional domain enrichment score with position-specific physicochemical properties (PSPCP). Li et al. (2015) proposed to fuse the position-specific scoring matrix (PSSM) and gene ontology to extract feature sequences.

Protein sequences often have high feature dimensionality and contain a large amount of redundant information, which reduces the prediction performance of a model. Dimensionality reduction of feature vectors with high-dimensional data is performed to eliminate unnecessary features. The cmmonly used methods are principal component analysis (PCA) (Ahmad et al., 2016), information gain (Wen et al., 2016), maximum correlation and minimum redundancy (MRMR) (Khan et al., 2017), maximum correlation maximum distance (MRMD) (Zou et al., 2016), singular value decomposition (SVD) (Silvério-Machado et al., 2015), local linear discriminant analysis (Yu et al., 2018), and dipeptide composition (DPC) (Ahmad et al., 2016). Xie et al. (2021) proposed a method for k-size optimal parsimony features based on the rough set theory, which found the effective features by fixing the parsimony size and dynamic weighting strategy. NMFBFS reduced the dimensionality of the data by decomposing the non-negative matrix of the data (Ji et al., 2015). Despite the specific advantages of existing methods for PVP prediction, there is a need to improve the accuracy and transferability of predictive models.

In the present study, we propose an ensemble learning-based feature selection method for phage virus protein classification that uses a four-step pipeline for protein prediction, (I) extracting the amino acid composition content (AAC), physicochemical properties CTD, dipeptide composition CKSAAP, and reduced position specificity scoring matrix (RPSSM) of the protein; (II) using ensemble learning to measure the importance of each feature component from each type of feature; (III) using an incremental strategy to select the most important feature subset; and (IV) combining the optimal feature subset derived from each type of feature to retrain the data to be filtered again and finally applying the obtained optimal feature subset to predict the protein type. Instead of the PCA-like feature dimension reduction method, our method can directly obtain important features for further biological analysis. Experimental results demonstrate the effectiveness of our method.

## Materials and Methods

### Data

In this study, we used the dataset constructed by Ding et al. (2014). This dataset was processed by UniProt in the following ways. First of all, the phage proteins whose subcellular location is a virion were considered positive sample and *vice versa*. The sequences containing unknown amino acids such as “B,” “J,” “O,” “U,” “X,” or “Z” were removed. To eliminate the influence of homologous sequences, more than 40% homologous sequences were removed by CD-HIT. Eventually, 99 phage proteins and 208 non-phage proteins were obtained.

The data from various literature studies (Feng et al., 2013; Ding et al., 2014; Zhang et al., 2015) were processed in the same way to build an independent test set. Besides, more than 40% homologous sequences with the training set were removed.

### Feature Extraction

The functions of a protein consists of the amino acid type, quantity, arrangement order of the peptide chain, and the spatial structure of the protein. Therefore, the main description methods of a protein can be divided into the global description of the protein and the description of the amino acid level (Xu et al., 2020). The global description of the protein includes the first-class features and spatial features of the protein. However, the acquisition of spatial features is expensive. Under certain conditions, the primary structure of the protein can determine the secondary, tertiary, and quaternary structures. Therefore, in this study, we only extracted the primary features of the protein and also extracted simple spatial structural features from the primary structure.

For the convenience of discussion, we define a protein sequence P as follows:


(1) $$\begin{array}{r} P=p_{1}p_{2}p_{3}\ldots p_{L},p_{i}\;\in \left\{A,\;C,\;D,\;\ldots Y\right\} \end{array}$$

where *p*_*i*_ is an amino acid, *i* is the position of the amino acid in the sequence, and *L* is the length of the amino acid sequence.

#### AAC

The Amino Acid Composition (AAC) is to calculate the content of each amino acid in a protein sequence. The AAC feature of an amino acid sequence is as follows:


(2) $$\begin{array}{r} AAC_{i}=\frac{N\left(p_{i}\right)}{L},\;0<i\leq 20 \end{array}$$

where *AAC*_*i*_ represents the proportion of *p*_*i*_ in a protein sequence, *N*(*p*_*i*_) is the number of the amino acid *p*_*i*_ in a protein sequence, and *L* is the number of amino acids in a sample.

#### CTD

Protein-related chemical reactions commonly occur in the cell and tissue fluids, so the physicochemical properties of a protein are closely related to the function of the protein. There are eight types commonly used with the following physicochemical properties: hydrophobicity, polarity, surface tension, polarizability, charge, van der Waals force, secondary structure, and solubility (Cai et al., 2003).

For each physicochemical property, the amino acids are divided into three groups (positive, neural, and negative), and then, the three values of Composition (C), Transition (T), and Distribution (D) of each property are calculated. C is the percentage of the composition of a certain physicochemical property. T describes the following three types of residue pairs: a negative residue followed by a neural residue; a positive residue followed by a negative residue; and a positive followed by a neural residue. The percentage of the amino acids of a particular property located at the first, 25, 50, 75, and 100% is measured as the distribution of the protein (D). Finally, 168 physicochemical features are obtained.

#### CKSAAP

The composition of k-space amino acid pairs (CKSAAP) is encoded by the proportion of amino acid pairs separated by any k residues.


(3) $$\begin{array}{r} CKSAAP=\frac{N_{p_{i}p_{j}}}{L},p_{i},p_{j}\;\in \left\{A,\;C,\;D,\;\ldots ,\;Y\right\}\\ j=i+k+1,\;i,\;j\leq L, \end{array}$$

where *N*_*p*_*i*_*p*_*j*__ is the proportion of residue pairs *p*_*i*_ and *p*_*j*_. According to Ding et al. (2014), the best results of extracting phage protein features for classification are obtained when *k* = 1.

#### RPSSM

In the process of biological evolution, amino acid sequences mutate corresponding to common changes, including deletion, substitution, and insertion of amino acid residues. However, these changed protein sequences still have similar structures and functions (Ding et al., 2014). The position-specific scoring matrix (PSSM) can describe this change.

For the convenience of description, we defined the sequence *P*_*A*_ as follows:


(4) $$\begin{array}{r} P_{A}={\left(P_{1A},P_{2A},\;\ldots \;,\;P_{LA}\right)}^{T} \end{array}$$

where *P*_*iA*_ indicates the score of amino acid mutation to amino acid A at the *ith* position of the protein sequence.

According to the similarity of amino acids, Li et al. (2003) found that 10 residues can construct a set with the smallest reasonable folding model, i.e., {F, Y, W}, {M, L}, {I, V}, {A, T, S}, {N, H}, {Q, E, D}, {R, K}, {P}, {C}, and {G}. We combined the same categories based on the similarity of amino acids as follows:


(5) $$\{\begin{array}{c} P_{1}\;=\frac{\left(P_{F}\;+\;P_{Y}\;+\;P_{W}\right)}{3},\\ P_{2}=\frac{\left(P_{M}\;+\;P_{L}\right)}{2},\\ \ldots ,\\ P_{10}=P_{G}. \end{array}$$

The reduced position-specific scoring matrix (RPSSM) is represented as follows:


(6) $$PSSM_{S}=\left[\begin{array}{ccc} \begin{array}{cc} P_{1,1} & \;P_{1,2}\\ P_{2,1} & \;P_{2,2} \end{array} & \;\begin{array}{c} \cdots \\ \cdots \end{array} & \;\begin{array}{ccc} P_{1,j} & \;\cdots & \;P_{1,10}\\ P_{2,j} & \;\cdots & \;P_{2,10} \end{array}\\ \vdots & \;\vdots & \;\vdots \\ \begin{array}{cc} P_{i,1} & \;P_{i,2}\\ \vdots & \;\vdots \\ P_{L,1} & \;P_{L,2} \end{array} & \;\begin{array}{c} \cdots \\ \vdots \\ \cdots \end{array} & \;\begin{array}{ccc} P_{i,j} & \;\cdots & \;P_{i,10}\\ \vdots & \;\vdots & \;\vdots \\ P_{L,j} & \;\cdots & \;P_{L,10} \end{array} \end{array}\right].$$

The variance of each column is calculated to get the feature *D*_*s*_,


(7) $$\begin{array}{r} D_{S}\;=\frac{1}{L}\;\overset{L}{\underset{i=1}{\sum }}{\left(p_{is}\;-{\bar{p}}_{s}\right)}^{2},\;s\leq 10,\;i\leq L \end{array}$$

However, *D*_*S*_ does not contain the amino acid order information. To get the information on the local sequence order effect, the dipeptide composition of the protein sequence is extended to PSSM. Assuming that *p*_*i*_ is mutated into *p*_*s*_ and that *p*_*i*+1_ is mutated into *p*_*t*_ in the sequence, then


(8) $$\begin{array}{r} D_{i,i+1}={\left(p_{i,s}-\frac{p_{i,s}+p_{i+1,t}}{2}\right)}^{2}+{\left(p_{i+1,t}-\frac{p_{i,s}+p_{i+1,t}}{2}\right)}^{2}\\ \;={\frac{\left(p_{i,s}-p_{i+1,t}\right)}{2}}^{2} \end{array}$$

where *s, t* = 1, 2, …10, *i* = 1, 2, … *L* 1, and *D*_*i, i*+1_ represents the difference between the average values of *p*_*i, s*_ and *p*_*i*+1, *t*_, while *D*_*i, i*+1_ in the protein sequence is expressed as follows:


(9) $$\begin{array}{r} D_{s,t}=\frac{1}{L}\overset{L-1}{\underset{i-1}{\sum }}\frac{{\left(p_{i,s}-p_{i+1,t}\right)}^{2}}{2},\;s,\;t=1,2,\ldots ,\;10 \end{array}$$

Finally, 110 features were obtained by splicing *D*_*s, t*_ and *D*_*s*_.

### Feature Selection

The goal of the feature-selection module is to select as few features as possible with guaranteed classification accuracy so that the model does not degrade significantly when the model is learned using only the subset of features and the learning results are close to or higher than the learning results using the full set of features.

The relationship between the sequence structure and the function of protein is not entirely clear. Therefore, the features based on knowledge extraction are not necessarily related to the function of the protein or are even some irrelevant features. Redundancy will also affect the fitting of the classifier to protein data and will interfere with the prediction. Therefore, we cannot select features only in a knowledge-driven way. It is necessary to further screen the extracted features in a data-driven way so as to screen out effective feature subsets beneficial to the learning algorithm. This can not only reduce the difficulty of learning tasks but also improve the efficiency of the model. We assume that only some of all the features play a decisive role in model fitting. If this feature is modified, it will have a greater impact on the results. To quantify this effect, we proposed a feature selection model. The specific steps we followed were as follows.

#### Importance Accumulation

**Figure 1:**
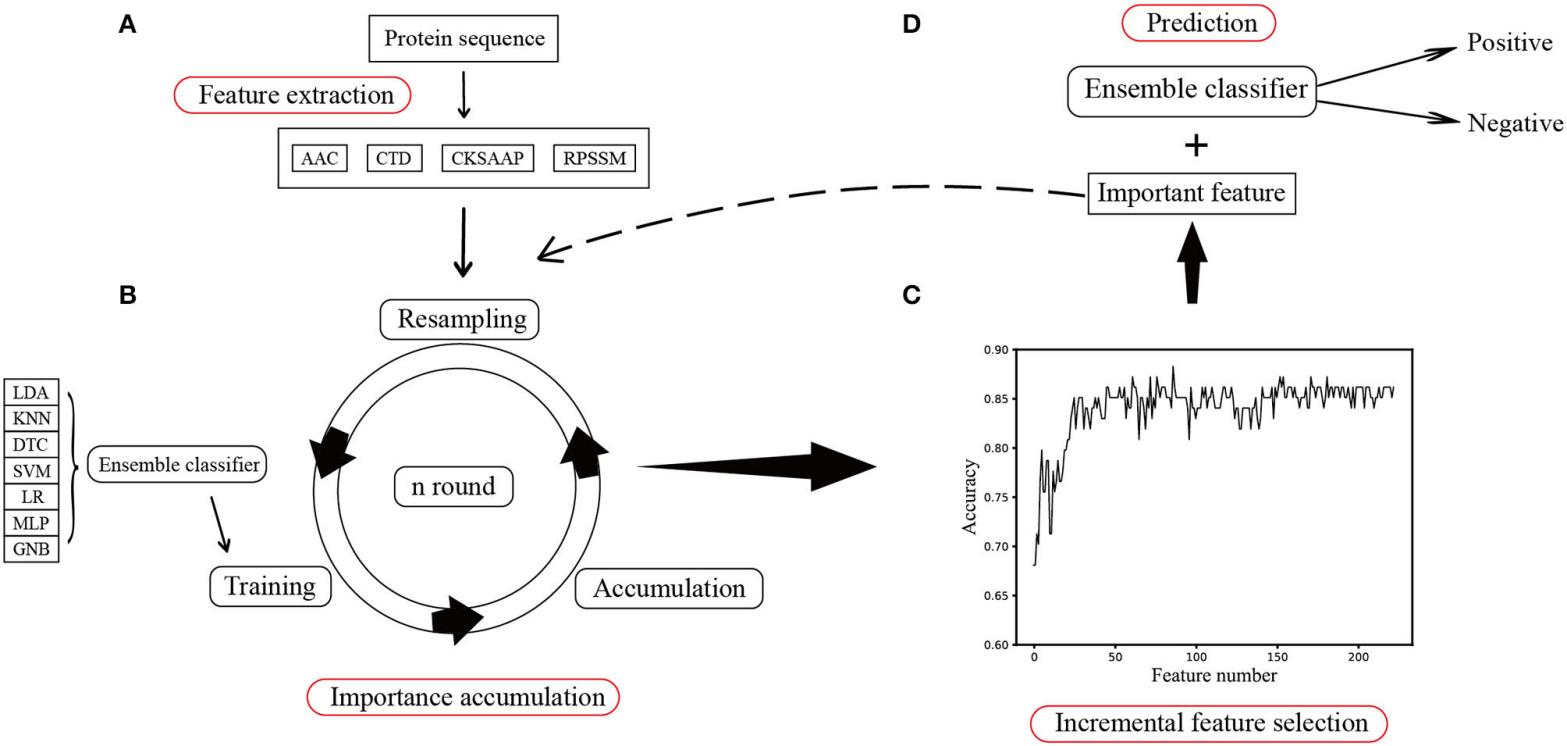
Flowchart of the proposed method. **(A)** Feature extraction method includes extracting AAC, CTD, CKSAAP-1GAP, and RPSSM from the original protein sequences. **(B)** Resampling all samples and then calculating the importance of each feature component using the ensemble classification. **(C)** Selecting a subset of important features using the incremental method. **(D)** Predicting phage proteins on the testing set using the important feature components.

First, the features from four types of protein sequences were extracted by a feature extraction module in Figure 1A. Then, the importance score of each feature component was calculated. According to the feature selection module shown in Figure 1B, we assumed that all the features extracted are valid for classification. Then, 70% of the training samples were randomly selected to train the classifier, and the classification accuracy rate was obtained by testing the data out-of-bag, which is recorded as *score*_1_. If a feature component was very relevant to the protein function, just changing it would have a great impact on the result. According to this idea, we only shuffled the values of a feature component randomly by a permutation way on the testing dataset in order to maintain the data distribution unchanged before and after this modification, then used the model trained on the training set to predict the shuffled data, and obtained the classification accuracy, which is recorded as *score*_2_. The importance of this feature component is defined as:


(10) $$\begin{array}{r} imp_{i}=score_{1}-score_{2} \end{array}$$

Each feature component is shuffled to get its important score. To reduce the error, it repeats n rounds to get the average importance scores of each feature.

#### Incremental Feature Selection

To find the optimal feature subset, we added each feature component incrementally according to its score in descending order, trained the classification model, and calculated the classification accuracy and finally showed the result in Figure 1C. In this way, a feature subset that maintains a comparative classification result equivalent to the feature is selected and considered an important feature. Then, the classifier was used in a feature subset to train all the training samples, and the independent test set was used to predict the phage protein. The final processed was shown in Figure 1D.

#### Ensemble Classifier

Due to the unclear sample distribution and various classification boundaries, only using a single classifier may not fit the data well and may not get a good classification result. Therefore, we used seven common classifiers with default parameters as the base classifier in scikit-learn (Pedregosa et al., 2011), linear discriminant analysis (LDA), decision tree classifier (DTC), k-neighbors classifier (KNN), support vector machine (SVM), logistic regression (LR), Gaussian Naive Bayes classifier (GNB), and multilayer perceptron (MLP) using 1,000 iterations. At the same time, seven classifiers were trained in each sampling, and the classifier with the highest accuracy was selected. We believed that this classifier can best fit the distribution of the data. This classifier was used as the best classifier in this round to classify or calculate feature importance. Although the ensemble learning method can be insensitive to the distribution of data, the time cost will increase due to the use of multiple classifiers. The time complexity of the proposed method $O\left(rounds*\underset{i}{\sum }classifier_{i}\right)$ was mainly to calculate the importance score of features, where rounds refer to the number of iterations. Therefore, if the time complexity needs to be reduced, the preferred method is to choose a classifier with smaller time complexity.

### Evaluation Criteria

To evaluate our model comprehensively, we used the common measures, i.e., ACC, SN, and SP. These methods are defined as follows:


(11) $$\begin{array}{rcl} ACC & = & \frac{TN+TP}{TP+FN+\;FN+\;FP} \end{array}$$

(12) $$\begin{array}{rcl} SN & = & \frac{TP}{TP+FN} \end{array}$$

(13) $$\begin{array}{rcl} SP & = & \frac{TN}{TN+FP} \end{array}$$

where TP, FP, TN, and FN denote true positive, false positive, true negative, and false negative, respectively.

## Results

The function of the protein consists of amino acid composition, arrangement order, and spatial structure. In this study, we selected amino acid content (AAC), physicochemical properties (CTD), dipeptide (CKSAAP-1GAP), and PSSM matrix of protein (RPSSM) as the features of protein data. The classification accuracy of each feature was calculated using the ensemble classifier to get the importance of each feature component. According to the importance of feature components, the effective feature subset of each feature of different species was selected in an incremental way. Finally, all feature subsets were spliced and predicted on the independent test set and compared with other methods.

### Performance Comparison of Individual Types of Features on Training Dataset Results Using Independent Features

**Figure 2:**
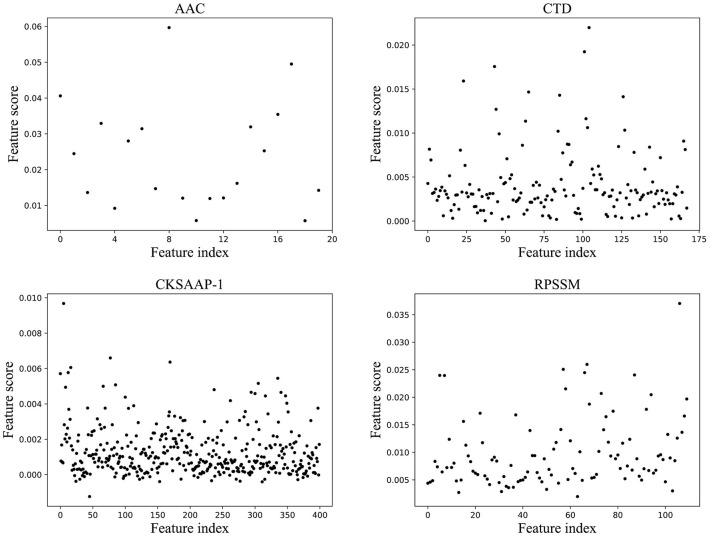
The scatterplots of feature importance using different feature extraction methods.

For features of different types, each classification model in the ensemble classifier was trained using the training dataset, and the model with the best fitting effect was selected by ACC in the ensemble classifier. The feature importance score was calculated using a feature selection module shown in Figure 1B. Figure 2 shows the scatterplots of the accumulated scores according to feature components. It can be found that AAC can affect the results by 6% at the highest and the physicochemical properties (CTD) by 2%, but dipeptide can only affect the results by 1% at the highest. RPSSM has an impact of 3.5%. Some features have no effect on the results after modification.

**Figure 3:**
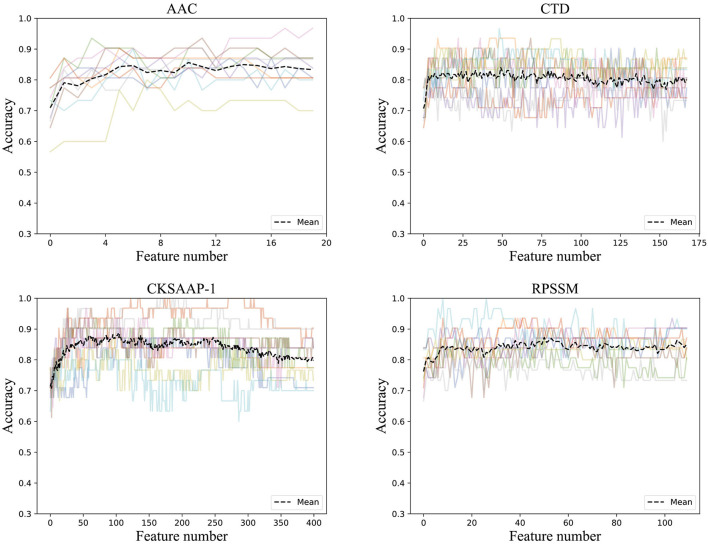
Accuracies derived from the incremental strategy using different feature extraction methods (The colored line shows the accuracy results of the 10-fold data).

To get rid of the ineffective feature components, we used the incremental method according to the rank of feature importance scores. After stacking feature components according to their importance, we trained the ensemble classifier and obtained the classification accuracies. The ACC curve was obtained using a 10-fold cross-validation method, as seen in Figure 3.

It can be found that the classification accuracy of the model in AAC has shown an increasing trend with the superposition of feature components, but it is around 82%. The accuracy of physicochemical properties (CTD) is more stable when features are superimposed. It can be found that the classification accuracy is the highest using only the first 50 important features, and the classification accuracy does not increase when feature components are superimposing. The classification accuracy of the first 103 feature components in CKSAAP-1GAP is also good with the highest classification accuracy, and the classification accuracy even decreases when new feature components are added. RPSSM is always more stable, and the first 50 feature components with the highest classification accuracy are selected as the optimal feature subset of the features.

### Performance Comparison of Concatenated Features on Training Data Set Results Using Integrated Features

We selected the subset of features with the best classification result and the least number of features among different kinds of features and spliced the obtained subset of features to form an important feature with multiple types of superpositions.

**Figure 4:**
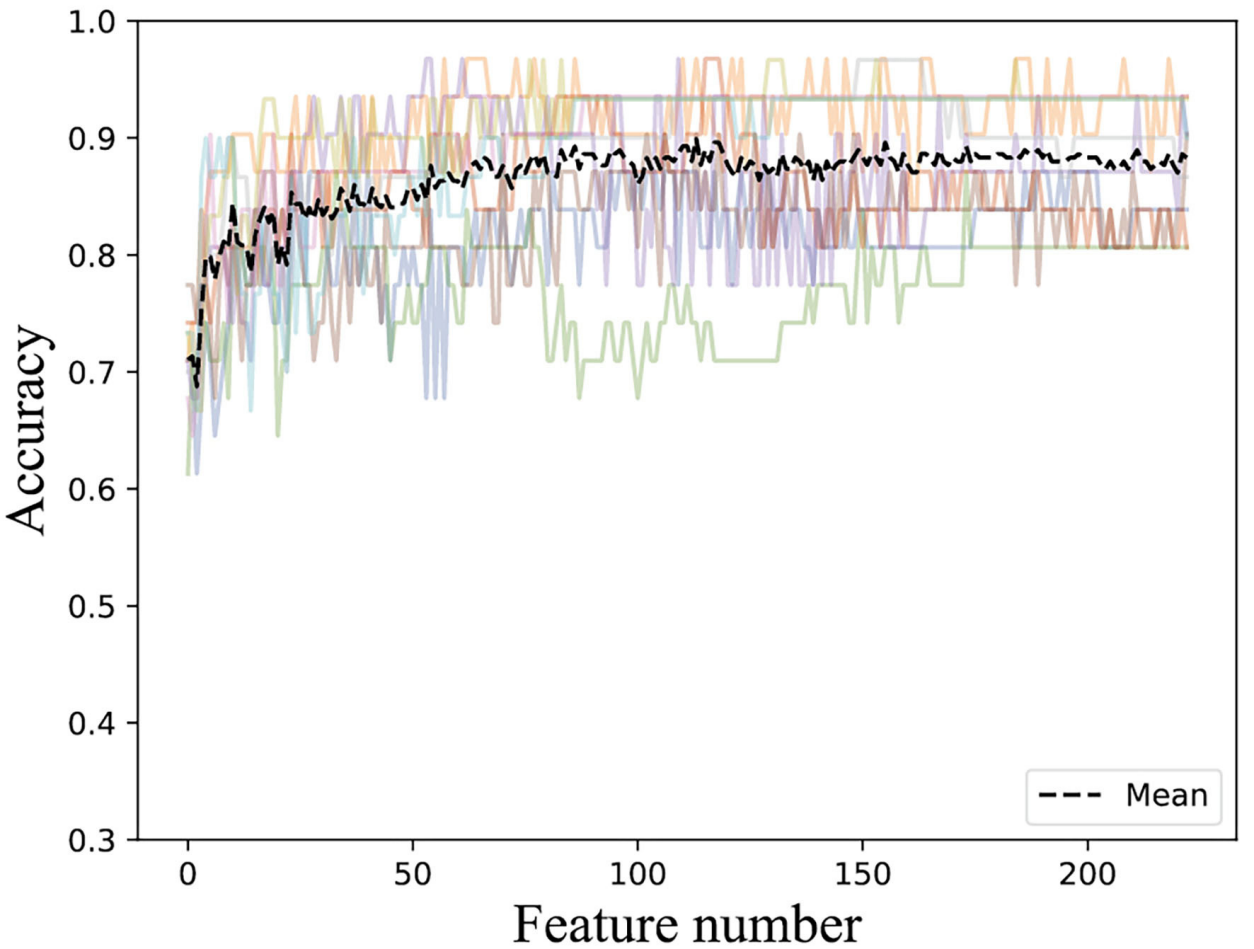
Accuracies derived from the incremental strategy using integrated features.

According to the important feature subset selected from the AAC sequence feature, the physicochemical feature, dipeptide 1-gap content, which has been indicated to be the best (Ding et al., 2014), and RPSSM feature, we obtained 223 feature components totally. Accordingly, the average classification accuracy was calculated in turn on the training set using a 10-fold cross-validation. The qualitative results are shown in Figure 4. The quantitative results compared with other methods are listed in Tables 1, 2.

**Table 1:** Average accuracy of a 10-fold cross-validation on the training set using different features.

**Classifier**	**Features**	**SN (%)**	**SP (%)**	**ACC (%)**
DTC, GNB, LR, MLP	AAC (20D)	71.89	85.02	80.81
MLP, KNN, DTC, LR	CTD (168D)	69.00	86.12	80.41
KNN, MLP, LR, GNB	CKSAAP_1gap (400D)	70.78	80.71	77.51
GNB, LDA, LR, MLP	RPSSM (110D)	82.78	79.81	80.81
MLP, GNB, LR, DTC, KNN	Concatenation (698D)	56.67	90.63	79.79
GNB, MLP, KNN, LR	**CTD (50D)**	**73.88**	**89.40**	**84.29**
GNB, KNN	**CKSAAP_1gap (103D)**	**82.06**	**88.07**	**86.04**
LR, MLP, DTC, GNB, LDA	**RPSSM (50D)**	**81.78**	**87.05**	**85.37**
DTC, LR, MLP, LDA, KNN	**Concatenation (38D)**	**83.00**	**85.05**	**86.00**
GNB, LDA, LR	**Concatenation (87D)**	**89.00**	**86.55**	**89.28**

*Bold indicates the result of the processing of the features*.

**Table 2:** Accuracy on independent test sets using different kinds of features.

**Classifier**	**Features**	**SN (%)**	**SP (%)**	**ACC (%)**
MLP	AAC (20D)	66.67	85.94	79.79
MLP	CTD (168D)	70.00	79.69	76.60
GNB	CKSAAP_1gap (400D)	63.33	85.94	78.72
GNB	RPSSM (110D)	73.33	76.56	75.53
**GNB**	**Concatenation (698D)**	56.67	90.63	79.79
**LDA**	**CTD (50D)**	**70.00**	**82.81**	**78.72**
**SVM**	**CKSAAP_1gap (103D)**	**46.67**	**92.19**	**77.66**
**MLP**	**RPSSM (50D)**	**60.00**	**92.19**	**81.92**
**MLP**	**Concatenation (38D)**	**73.33**	**90.63**	**85.11**
**MLP**	**Concatenation (87D)**	**73.33**	**93.75**	**87.23**

*Bold indicates the result of the processing of the features*.

It can be seen from the 10-fold results that the classification accuracy of only the first 20 feature components of AAC is better than that of the others. On the independent test set, the classification accuracy rate is 79.79%. It shows that the amino acid content of phage protein is quite different from that of the non-bacterial protein in these data, especially lysine and valine are the most important residues. The classification accuracy of CKSAAP-1GAP is not as good as that of AAC. Because the number of the feature subsets is 30% more than the total number of training samples, the classification accuracy rate is increased to 86.04% when 103 important feature components are selected, but the classification accuracy rate is only 77.66% when only CKSAAP-1GAP is used in the independent test set. However, the classification results of other individual feature subsets also show that the classification accuracy of only using any individual feature subsets is always low, so we decided to splice different types of feature subsets. To prevent the dimension from being too high, we only selected important features to combine.

For the training set of 223 dimensional features after splicing, we calculated the feature importance again using the feature selection modules shown in Figure 1B to calculate the influence of each feature on the results when different feature components were spliced. The features were then further filtered in the training data set using the incremental approach and cross-validation at 10-fold.

**Figure 5:**
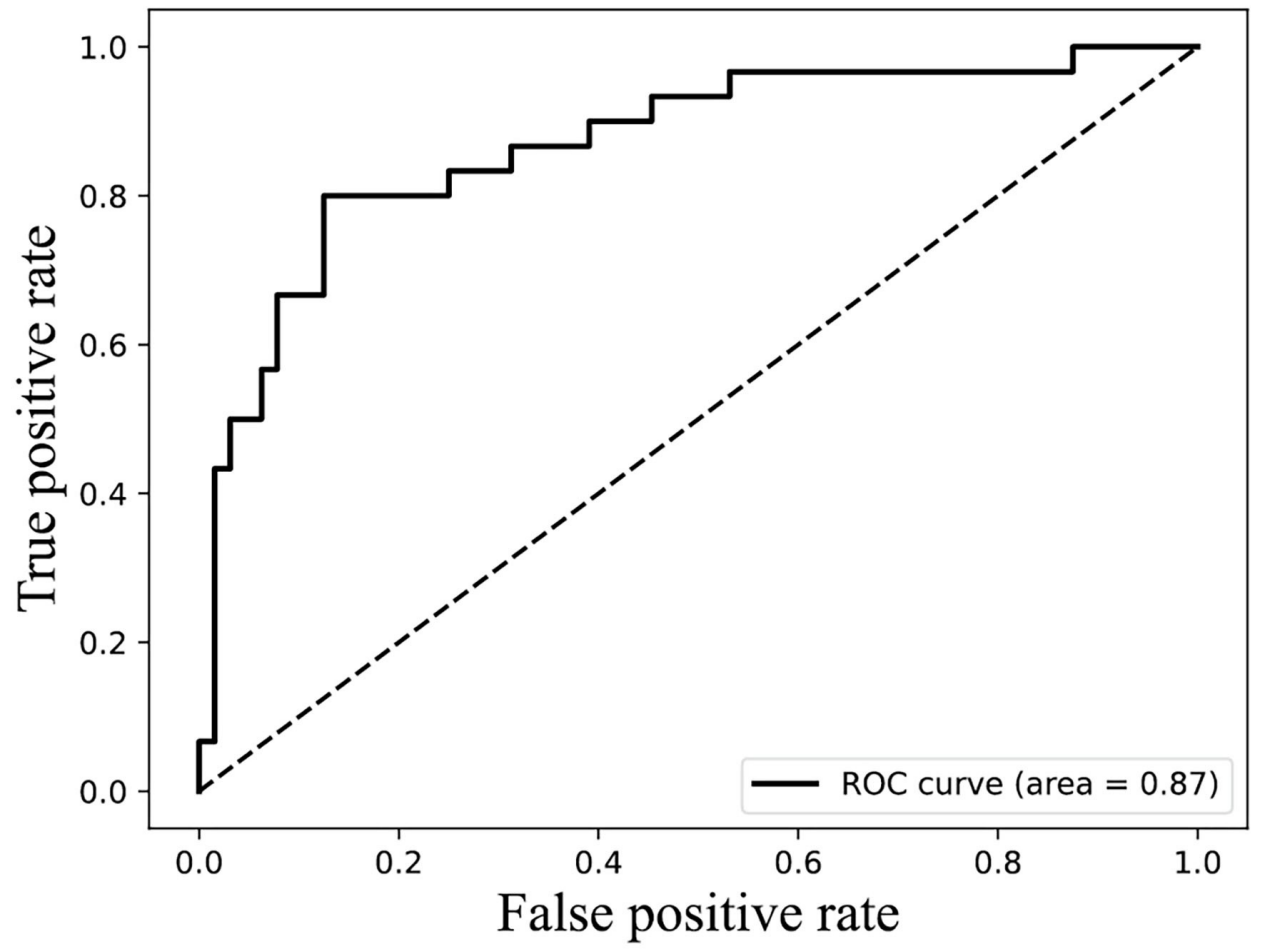
ROC curves of 87 features on independent test data.

The result of Figure 4 shows that 85% classification accuracy, which is the same classification accuracy as stated in the original article (Ding et al., 2014), can be achieved on the independent test set with 55 feature components selected, and 89.28% on the training set and 86.17% on the independent test with 87 feature components selected. Although the accuracy is 0.65% higher than 87 at the 113-dimension level, the number of feature components is greatly increased. Therefore, we chose 87 features for prediction on the independent test set, and the ROC curve is shown in Figure 5.

From Table 1, it can be seen that the classification accuracy is low using only one type of all features and that using feature selection is significantly higher. As can be seen from Table 3, we can achieve the same results with fewer feature components compared to the original article (Ding et al., 2014).

**Table 3:** Performance comparison of the different features in independent test sets.

**Classifier**	**Features**	**SN (%)**	**SP (%)**	**ACC (%)**
Naïve Bayes	Ding et al., 2014 (38D)	75.76	80.77	79.15
SVM	Ding et al., 2014 (160D)	75.76	89.42	85.02
Bin et al., 2020	Nine feature groups (8D)	50.00	92.19	78.72
**MLP**	**Concatenation (38D)**	**73.33**	**90.63**	**85.11**
**MLP**	**Concatenation (87D)**	**73.33**	**93.75**	**87.23**

*Bold indicates the result of the processing of the features*.

For the 17 selected 1-gap dipeptides (A^*^G, A^*^T, A^*^P, S^*^T, S^*^A, V^*^A, T^*^S, V^*^T, G^*^A, G^*^G, S^*^G, V^*^G, V^*^I, E^*^L, K^*^L, K^*^E, and E^*^E) in the original article (Ding et al., 2014), the order of feature importance is (1, 4, 5, 17, 15, 14, 7, 61, 18, 25, 8, 161, 16, 23, 3, 87, and 54) when determined individually. It can be found that there is a significant overlap between the feature we selected and the original article (Ding et al., 2014). Lin et al. analyzed the amino acid composition of filamentous bacterial virus xf (*Xanthomonas oryzae*) coat protein, which showed that His, Cys, and Phe were absent from the xf protein. This indicates that these three amino acids are not important in phage classification. In AAC features, the rankings of these three amino acids are 7, 9, and 17. It also occupies 1, 0, and 1 amino acids in the top 20 CKSAAPs.

## Discussion and Conclusion

By analyzing the selected feature, it can be found that physicochemical properties are important for phage protein identification. In fact, 40 components representing physicochemical properties appear in the 87 features spliced. Of the first 15 features, 12 refer to the physicochemical properties. The most important feature comes from the physicochemical properties. The charge property is the most important, followed by polarity and polarization rate. Besides, the effective physicochemical properties are derived from different feature extraction methods. The contribution of CKSAAP-1GAP to the 87-dimensional feature is limited. Only 9 of CKSAAP-1GAP features are selected, while the secondary structure composed of the physicochemical properties derived from CTD occupies 20. Thus, it can be concluded that CTD makes a greater contribution than CKSAAP-1GAP on the selected feature for the classification of phage protein. As to AAC, its most important components are proline and leucine, which are highly ranked in the 87 features spliced. For RPSSM, its selected features are not top ranked in the 87-dimensional feature, but 30 features appear in the 87 features spliced. The extracted secondary structures using CTD and RPSSM after classifying amino acids in advance according to their properties are highly ranked in the 87 feature components, while the ones derived from CKSAAP-1GAP are not.

In this study, a feature selection framework is proposed for the protein classification of phages. The model improves the classification accuracy of the data by overlaying different types of features. To prevent overfitting caused by high feature dimensionality, the feature importance was quantified and the important features with high scores were selected as the final feature for classification. We performed ensemble learning using different classifiers, which are insensitive to the distribution of the original data, quantified the importance of each feature, and then performed feature selection on it. Finally, only an 87-dimensional feature was used to achieve a high classification accuracy. Compared with the original article (Ding et al., 2014) and PredNeuroP (Bin et al., 2020), a new method for recognizing phage protein, the model can achieve the same classification accuracy using only 38 feature components. The classification accuracy reaches 87.23% when the optimal 87-dimensional feature is used. It also shows that, when the relationship between the structure and function of phage proteins is not fully understood, the knowledge-driven approach to feature extraction alone does not necessarily lead to better prediction results. In contrast, the prediction of phage proteins by a combination of knowledge-driven and data-driven is more accurate, and the functions of phage proteins can be further investigated by analyzing the selected feature.

## Data Availability Statement

The original contributions presented in the study are included in the article/supplementary material, further inquiries can be directed to the corresponding author/s.

## Author Contributions

SL and CC proposed this research topic. TL completed the experiment and wrote the manuscript. HC supervised the experimental process and reviewed the manuscript. All authors contributed to the article and approved the submitted version.

## Conflict of Interest

The authors declare that the research was conducted in the absence of any commercial or financial relationships that could be construed as a potential conflict of interest.

## Publisher's Note

All claims expressed in this article are solely those of the authors and do not necessarily represent those of their affiliated organizations, or those of the publisher, the editors and the reviewers. Any product that may be evaluated in this article, or claim that may be made by its manufacturer, is not guaranteed or endorsed by the publisher.
